# OCDB: a database collecting genes, miRNAs and drugs for obsessive-compulsive disorder

**DOI:** 10.1093/database/bav069

**Published:** 2015-07-29

**Authors:** Anna P. Privitera, Rosario Distefano, Hugo A. Wefer, Alfredo Ferro, Alfredo Pulvirenti, Rosalba Giugno

**Affiliations:** ^1^Department of Clinical and Experimental Medicine, University of Catania, Viale A. Doria 6, Catania, Italy,; ^2^Istituto di Scienze Neurologiche, CNR, Via Paolo Gaifami, 18, 95125 Catania, Italy,; ^3^Department of Computer Science, University of Verona, Strada le Grazie 15, Verona, Italy and; ^4^KarolinskaInstitutet, Department of Microbiology, Tumor and Cell Biology, Science for Life Laboratory, Stockholm, Sweden

## Abstract

Obsessive-compulsive disorder (OCD) is a psychiatric condition characterized by intrusive and unwilling thoughts (obsessions) giving rise to anxiety. The patients feel obliged to perform a behavior (compulsions) induced by the obsessions. The World Health Organization ranks OCD as one of the 10 most disabling medical conditions. In the class of Anxiety Disorders, OCD is a pathology that shows an hereditary component. Consequently, an online resource collecting and integrating scientific discoveries and genetic evidence about OCD would be helpful to improve the current knowledge on this disorder. We have developed a manually curated database, OCD Database (OCDB), collecting the relations between candidate genes in OCD, microRNAs (miRNAs) involved in the pathophysiology of OCD and drugs used in its treatments. We have screened articles from PubMed and MEDLINE. For each gene, the bibliographic references with a brief description of the gene and the experimental conditions are shown. The database also lists the polymorphisms within genes and its chromosomal regions. OCDB data is enriched with both validated and predicted miRNA-target and drug-target information. The transcription factors regulations, which are also included, are taken from David and TransmiR. Moreover, a scoring function ranks the relevance of data in the OCDB context. The database is also integrated with the main online resources (PubMed, Entrez-gene, HGNC, dbSNP, DrugBank, miRBase, PubChem, Kegg, Disease-ontology and ChEBI). The web interface has been developed using phpMyAdmin and Bootstrap software. This allows (i) to browse data by category and (ii) to navigate in the database by searching genes, miRNAs, drugs, SNPs, regions, drug targets and articles. The data can be exported in textual format as well as the whole database in.sql or tabular format. OCDB is an essential resource to support genome-wide analysis, genetic and pharmacological studies. It also facilitates the evaluation of genetic data in OCD and the detection of alternative treatments.

**Database URL:** http://alpha.dmi.unict.it/ocdb/

## Introduction

Obsessive-compulsive disorder (OCD) is a neurological disease characterized and recognized by intrusive, persistent and unwanted thoughts (obsessions) and repetitive behavior (compulsions). It is a highly debilitating disease because the patients cannot live a normal existence, in fact they have to repeat the same actions or think the same thoughts to obtain a temporary relief (1, 2). The recurrent phenotypes are symmetry/ordering, hoarding, contamination/cleaning, and obsessions/checking (1) Another phenotype is the ‘pure obsessional’ with sexual, somatic, religious obsessions and mental rituals (2). In some instances, this behavior can include trying to avoid to walk on lines of sidewalks and floors; washing hands or face time after time; sorting objects by size; performing precise thoughts or prayers before entering in a place. OCD affects both children and adults (3, 4). In many cases, the first symptoms appear in childhood and early adolescence. Most of the patients are under the age of 20 (5), and in particular, 21% of the cases are children around the age of 10 years (6). Historically, the guidelines of International Classification of Diseases, 10th revision (ICD-10) and Diagnostic and Statistical Manual of Mental Disorders, 4th Edition, Text Revision (DSM IV TR) classified OCD into the anxiety disorders class (7, 8). The anxiety disorders were a broad and heterogeneous group of disorders including acute stress disorder, agoraphobia and many others (8). Recently, the American Psychiatric Association has published an upgrade in which OCD appears in a separate category called Obsessive-compulsive and related disorders (OCRDs) (9, 10). Thus, there is a growing attention for this disorder, with changes in the diagnostic criteria together with significant clinical implications (11). The World Health Organization, accordingly, ranks OCD as one of the 10 most disabling medical conditions worldwide (12).

OCD was described, for the first time, around a century ago by psychologist Pierre Janet (13). Nowadays, we know that OCD has a genetic component for its origin. The first fundamental OCD genome-wide linkage scan study was conducted by Hanna *et al*. (14) in which they identified regions in chromosomes 2, 9 and 16. Several other similar studies analysed the genetic linkage between 9p24 and OCD, and moreover, a few candidate genes have been identified (15, 16).

Few studies have highlighted the heritability of such a disease. In particular, the early onset of OCD (17) and the ordering/symmetry symptoms of OCD (18) are frequently heritable. A high risk of developing OCD or some symptoms in first-degree relatives has also been reported (19–21).

We know that in the 9p24 chromosomal region there is a candidate gene associated with OCD, *SLC1A1*/*EAAC1* (22, 23), which encodes for the neuronal/epithelial high affinity glutamate transporter (23). *SLC1A1* is expressed in some functional areas of the brain (24) and is implicated in OCD in the cortico-strial-thalamic-cortical circuits (CSTC) (25). Much evidence shows an altered glutamate neurotransmission within CTSC circuits in the pathophysiology of OCD (25). The neuronal glutamate transporter gene (*SLC1A1*) is mainly expressed within CSTC circuits (24). To date, researchers have identified many candidate genes as well as some regions of the genome that might include disease genes (26). Such genes include *SLC6A4* (27, 28), *HTR2A* (29, 30), *HTR2C* (29), *NTRK3* (31) and *SLITRK1* (32).

The nervous system requires an accurate regulation at all control levels. The post-transcriptional regulation of genes has a fundamental role in the pathophysiology of neurologic diseases (33); and for this reason, a great deal of research has been devoted to microRNAs (miRNAs) (34), which constitute a small class of non-coding RNAs. These molecules are ∼22 nucleotides (nt) long and are involved in several processes such as gene expression regulation, translational repression, mRNAs degradation and RNA silencing (35, 36). In addition, they also regulate >30% of all protein-coding genes (36). Muiños-Gimeno *et* *al*. (37) found a role of the neurotrophin-3 receptor gene (*NTRK3*) in the pathophysiology of anxiety disorders. In fact, such a gene has a high variation in the miRNA recognition element (MRE) of miR-485-3p, which is one of the most important miRNA associated to OCD (37). Finally, guidelines to treat OCD include both a psychotherapy called cognitive behavior therapy (CBT) and a pharmacological therapy. CBT is often not enough in ‘pure obsessional’ because patients have to control many rituals (38). The World Federation of Societies of Biological Psychiatry published the guidelines for the pharmacological treatment of OCD (39). Positive evidence is associated with the usage selective serotonin reuptake inhibitors (SSRIs) such as fluvoxamine, fluoxetine, escitalopram, paroxetine and sertraline, and for tricyclic antidepressant (TCA) such as clomipramine (39).

Despite the efficacy of some approved drugs (such as SSRIs and clomipramine), ∼40–60% of patients do not show any significant improvements (40, 41). Consequently, alternative treatments and new drugs in combination with SSRIs or TCA, might become necessary.

A study based on agomelatine augmentation to escitalopram therapy shows promising results (42). In fact, agomelatine is a 5-HT2c receptor antagonist (43) that determines a therapeutic response with total remission of the symptoms and with a Yale-Brown Obsessive-Compulsive Scale (Y-BOCS) (44) score of 5 (initial Y-BOCS score was of 33) (42).

In general, Y-BOCS evaluates the severity of OCD symptoms using a score ranging from 0 and 40. In fact, OCD scores can be 0–7, sub-clinical; 8–15, mild; 16–23, moderate; 24–31, severe; 32–40, extreme (44).

In this work, we propose the first manually curated database, named OCD Database (OCDB), which collects genomic evidence related to OCD from literature. OCDB lists information about genes and chromosomal regions involved in the disease, which include transcription factors, regulations, polymorphisms, predicted and validated miRNAs, drugs targeting such genes, pathways in which genes enter, drugs currently used to treat OCD as well as the related pathologies. Moreover, we have defined a scoring method to assess the relevance to OCD of each item in OCDB. OCDB is equipped with a web interface in which users can search and browse genes, articles, miRNAs, Single-nucleotide polymorphisms (SNPs), drugs, drug targets and chromosomal regions related to OCD. OCDB returns the searched entities with all the information connected to them. Users can save the results in.html and.txt formats. The entire database is also provided in.sql and table formats. OCDB is available online at: http://alpha.dmi.unict.it/ocdb/.

## Materials and methods

OCDB is a relational database implemented in MySQL with a web interface that was developed using phpMyAdmin and Bootstrap software. The database is hosted at the University of Catania and is accessible at http://alpha.dmi.unict.it/ocdb/

**Figure 1.**
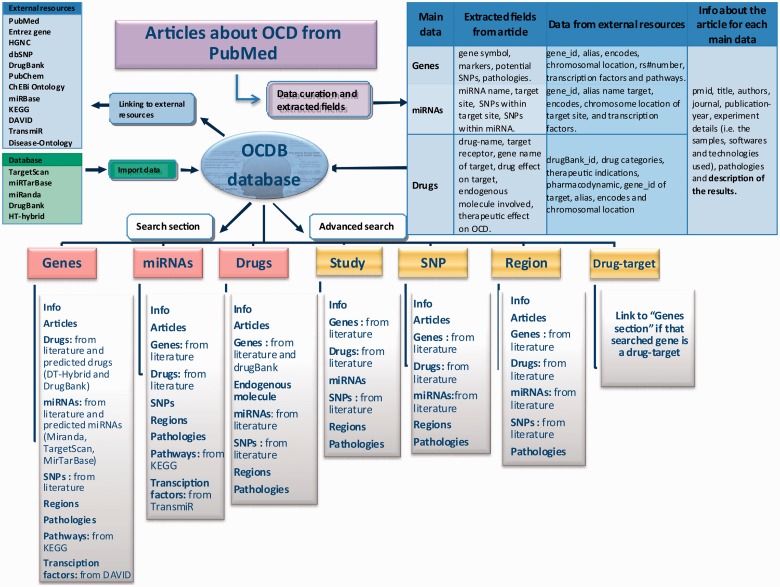
OCDB architecture.

Figure 1 reports the architecture of OCDB. It describes the data extracted from literature and other external resources, how they are linked and the users’ assess modes.

### The manually curated data in OCDB

The data stored in OCDB database has been collected from PubMed (45) and Medical Literature Analysis and Retrieval System Online (MEDLINE), updated until March 2015. By querying the earlier databases, we have done a comprehensive search concerning ‘OCD and genes, miRNAs, SNPs, drugs, drug therapy and treatments’, ‘OCD and genes’ and ‘OCD and genetic association studies’. This yield an outcome of 11 129 articles. Next, we have excluded the articles focusing on the psychological and epidemiological aspects, studies not in human and articles without an English abstract or completely lacking an available abstract. Finally, we obtained 1076 suitable articles for further processing.

A custum Python script, utilizing the Entrez API, helped us to retrieve data from National Center for Biotechnology Information (NCBI) resources (46). Through this, we obtained information about the articles (such as pmid, title, authors, journal, publication year) as well as gene symbols, SNPs and drug names, which were retrieved from Drugbank. In addition, OCDB includes manually curated data extracted from the articles. These include miRNAs names, experimental details (number of the samples, software and technologies used), notes about the scientific results, genomic regions, the role of the genes in the pathologies in comorbidity with OCD, the pharmacodynamics and pharmacological actions (if known) and the effects of the drugs and endogenous molecules on OCD.

The data available in the current version of the database cover 536 publications reporting on 180 polymorphisms in 153 genes and 16 miRNAs, 148 regions, 25 related pathologies as well as 48 drugs.

#### OCDB external resources.

To enrich its content, OCDB provides links to several external resources. These include (i) Entrez-gene (v. 2015-04-05)(GRCh38) (46) and HUGO Gene Nomenclature Committee (HGNC) (v.2.0.0 2015-01-19)(GRCh38) (47) to have official gene names and gene_ids; (ii) Single Nucleotide Polymorphism database (dbSNP) Build 143 (48) to retrieve the rs# numbers of SNPs; (iii) DrugBank (v.4.2) (49) to have information about drugs, such as pharmaceutical categories and therapy indications; (vi) miRBase(v.21) (50) and Pubchem (v. 2015-04-9) (51) to have more research material linked with miRNAs and endogenous molecules, respectively. In addition, transcription factors for genes and miRNAs are obtained from DAVID (v. 6.7) (52, 53) and TransmiR (v.1.2) (54). Genes have been associated to their respective pathways by using Kegg-Pathway (v.2015-04-06) (55, 56). Diseases and drugs have been linked to the disease-ontology (v. 2015-04-18) (57) and ChEBI ontology (v. 2015-04-01) (58), respectively.

#### Data prediction.

To help inferring new venues in the treatments and in the underlying genetics, miRNA-gene and drug-targeting predictions have been integrated within OCDB.

Notably, miRNA predictions have been obtained by using TargetScan (59), miRTarBase (60) and miRanda (61) while drug predictions have been obtained from DrugBank (49) and by using Domain Tuned-Hybrid (DT-Hybrid) algorithm (62). Predictions from DrugBank (49) are distinguished among Targets, Enzymes, Transporters and Carriers.

More precisely, the DT-Hybrid algorithm implements a functional framework, based on a recommendation technique, for the *in* *silico* prediction of drug-target interactions. It plugs into the network-based inference model specific domain knowledge such as the similarity among drugs and targets. The algorithm produces a set of new associations from which novel biological insight can be discovered. For each drug-predicted target pair, the algorithm also associates a score measuring the degree of certainty of the interaction. Such a value depends strongly on the neighborhoods of the drug and target, and their similarity to the neighbors. The range of each score is (0, 3), where zero indicates the absence of interaction, and three indicates a reliable interaction.

#### Scoring method.

We defined a score method to measure the relevance (specificity) of the information in our system to OCD. The concept of relevance of an item A (gene, miRNA, drug, SNP, region) to the pathology OCD is defined through a triplet of values normalized by z-scores (63):

- *w_1_*: the number of articles in OCDB containing *A* and the association with the pathology OCD.
- *w_2_*: the number of relations *(A, B)* in the database. If A is:
  - *gene;* then *B* is a miRNA or drug cited in the same article, a SNP or a region in *A*.
  - *miRNA;* then *B* is a gene or miRNA cited in the same article, a SNP or region in *A*.
  - *drug;* then *B* is a gene, miRNA, region or SNP cited in the same article.
  - *SNP:* then *B* is a gene, miRNA, region or drug cited in the same article.
  - *Region;* then *B* is a gene, miRNA, SNP or drug cited in the same article.
- *w_3_*: the number of articles in the database reporting the association among *A* and some other pathology, mentioned in the articles, related to OCD.

The score of *A* is the linear combination of *w_1_, w_2_* and *w_3_* normalized by z-score. The ratio behind this ranking is that OCDB does not highlight the biological importance of the item itself or the research involving it; but it weights how in our manually curated resource the item is specifically associated to OCD (w_1_) and to close other related pathologies (w_3_), and as much information about this item and its ‘neighbors’ (i.e. its relations) can be extracted from OCDB (w_2_). Consequently, the articles are not classified but instead the items they contain. Finally, the scores are also presented as classes according to the quartile they belong; the quartiles are represented by colors and numbers (red-1, yellow-2, green-3, light blue-4).

### Data interface

The main OCDB interface modules are Search, Advanced Search, Browse and Download/update. These allow (i) to browse the data; (ii) to navigate in the database by searching genes, miRNAs, drugs, articles, SNPs, regions and drug target; and (iii) to download and upload the data.

For each search type, OCDB guides the writing to avoid misspelled inputs. There are two levels of input control checking, a client side and a server side. The input nomenclature is based on the official scientific standard commonly used by online databases. The statistics on the OCDB data are reported together with a brief documentation on the usage of the database content.

#### Search and Advanced Search.

The Search section allows users to query the system by genes, miRNAs and drugs. In the Advanced Search, users can query OCDB by article, SNPs, regions and drug target. The drug target section points to genes that are targets of drugs used in the treatments.

Genes are specified by using the nomenclature of HGNC (49) and Entrez-gene by NCBI (46). The nomenclature of miRNAs refers to the ones used in miRBase (50). Drugs are inserted by their names as reported in DrugBank (49). Studies can be retrieved through a full text search by entering the title, the author names or the year of publication. SNPs should be inserted by rs# number corresponding to the nomenclature in dbSNP (48). If the user searches by region, the nomenclature references to cytogenetic standards.

**Figure 2.**
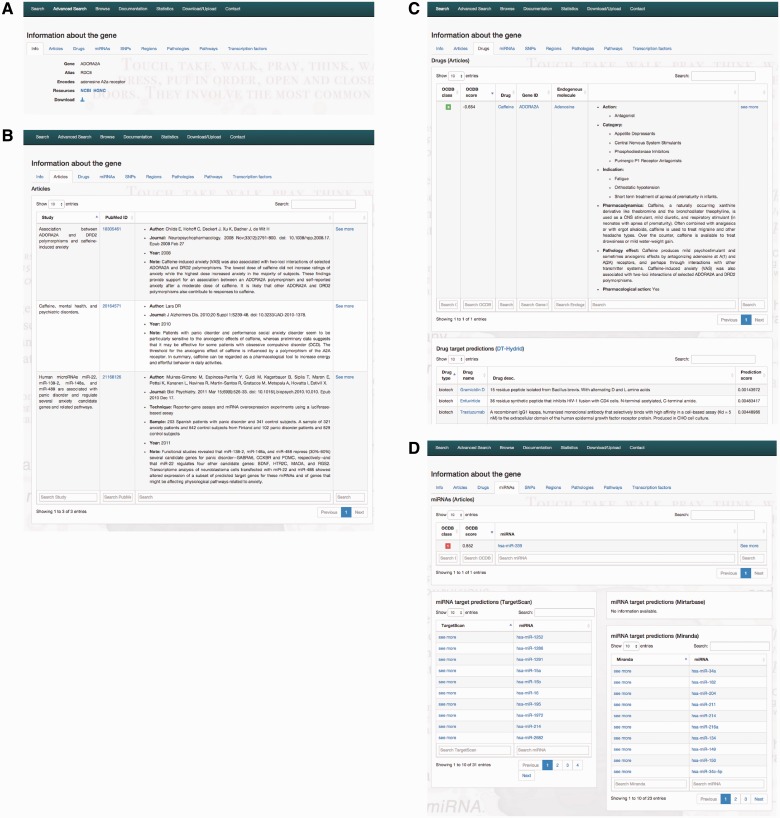
OCDB Interface. (**A**) General information about ADARO2A, links to HGCN and NCBI and the gene card download option. (**B**) Information about the articles mentioning results on ADARO2A are listed. (**C**) Drugs targeting ADARO2A taken from literature. The page reports also drugs targeting (not shown here) predicted by DT-Hybrid algorithm and taken from DrugBank. Other OCDB interface sections are not shown. (**D**) miRNAs mentioned in the articles targeting ADARO2A are given. Validated and predicted miRNAs from online databases targeting ADARO2A are listed.

Results are organized in the following cards (see Figure 2). We refer searched genes, miRNAs, drugs, articles, SNPs or genomic regions to as items.

- **Info:** Info gives nomenclature information, links to the web resources and allows downloading a full card of the searched item. If the item is an article, it shows details of the publication (i.e. the title, the author names, the journal, the publication year, note describing the results, if available details on the used techniques, sample and software) together with a link to PubMed (45). When the searched item is a gene, OCDB reports entrez_id, gene names, aliases, encodes and links to HGNC and NCBI. If the item is a miRNA, the system shows miRBase access number together with a link to the resource. If the item is a drug, OCDB reports the drug names, the drug pharmacodynamics and links to DrugBank and ChEBI. If the item is a SNP, it shows names and NCBI link. Finally, if it is a region, its name is given.
- **Articles:** Articles show references to articles mentioning the item (the title, the author names, the journal, the publication year, note describing the results) and the PMID linking to PubMed (45).
- **Genes:** Genes show the genes, which are targets of the searched miRNA or drug, containing the SNPs or the genes in the searched chromosomal region. It reports the links to HGNC and NCBI, alias, encodes and the role of genes (if the genes are retrieved from articles or if the genes are validated and/or predicted miRNA’s target).
- **Drugs:** If the item is a gene, shows the drugs that have the gene as a target, linked to DrugBank 4.2 (49) together with its type and a description, if available. The data are extracted from DrugBank (49) (targets, transporters and enzymes) or computed by the DT-Hybrid algorithm (62). For all other items, this section reports details on drugs (if any) that in OCDB articles have been associated with the searched item. Details are the gene names linked to Entrez by NCBI (46), endogenous molecules involved and the actions that the drugs has on the targets. There may also be information about the therapeutically indications, pharmacodynamics, pharmacological action and eventually the effects of drug on OCD extracted from the indexed articles.
- **Endogenous molecules**: if the item is a drug, it shows the endogenous molecules and genes on which the drug may have effect and their description. The effect is specified in ‘action’. The name of molecules is linked to PubChem (51).
- **miRNAs:** miRNAs show the miRNA-name linked to miRBase (50). If the item is a gene, it reported the prediction of miRNAs targeting such a gene made by TargetScan (59), miRanda (61) and mirTarBase (60).
- **SNPs:** SNPs show the SNPs present in the item. The SNP name is linked to dbSNP (48). If the item is not a gene, OCDB shows also the gene name linked to Entrez-gene card (46) or the miRNA access number containing the SNP.
- **Regions:** Regions give the chromosomal location of the selected item (i.e. a gene, miRNA, SNP); When the item is a drug, region yields the chromosomal location of targeted genes; When the item is a region, the list of gens, SNPs and miRNAs falling in such region are given together with the list of article citing such a region.
- **Pathologies:** Pathologies show pathologies in which the item is associated with OCD or with any other pathology related to OCD (such as social phobia or schizophrenia in comorbidity with OCD), which has been reported in literature. Each pathology is associated to Disease-ontology code (57).
- **Transciption factors:** By using DAVID (52, 53), we have obtained transcription factors related to our gene set. For miRNAs, we retrieved from TransmiR (54) the genes-miRNAs regulations. Note the update gene name of transcription is linked to Entrez as well as the gene-miRNAs regulation is linked to miRBase.
- **Pathways:** Pathways gives the list of pathways in which genes enter.

Users can visualize all details and relative relations with genetic elements present in the database of each item reported in the results cards by clicking ‘see more’. This is useful to navigate inside the database.

#### Browse.

This interface lists all genes, drugs, miRNAs, studies, SNPs and regions in OCDB. For all items, with the exception of studies, the relevance to the OCD is returned.

More precisely, when browsing for ‘Gene’, OCDB lists the gene name, its aliases and if it is a target of drugs used in OCD treatments. This section contains a tab to list all genes per chromosome. ‘Drug’ lists all names of drugs clinically used in OCD or related pathologies as reported in the articles in the OCDB. By clicking in ‘miRNA’ users can see the name and a description of the role of the miRNAs in the pathology. This is one of the sentences extracted from related articles. ‘Studies’ lists the year of publications, the title and PubMed-id. SNPs and Region-ids are listed in two distinct sections. ‘Regions’ are associated with markers, when available. In all browsing sections by clicking on ‘see more’, users can visualize the details and the relative relations with genetic elements present in OCDB.

The users can visualize the results in alphanumeric order with respect to any of the listed attributes. Moreover, a score representing the relevance of the gene, miRNA, drug, SNP or region with respect to the disease is given. For the earlier elements, users can order results in descending or ascending order by using such attribute scores.

#### Statistics.

**Figure 3.**
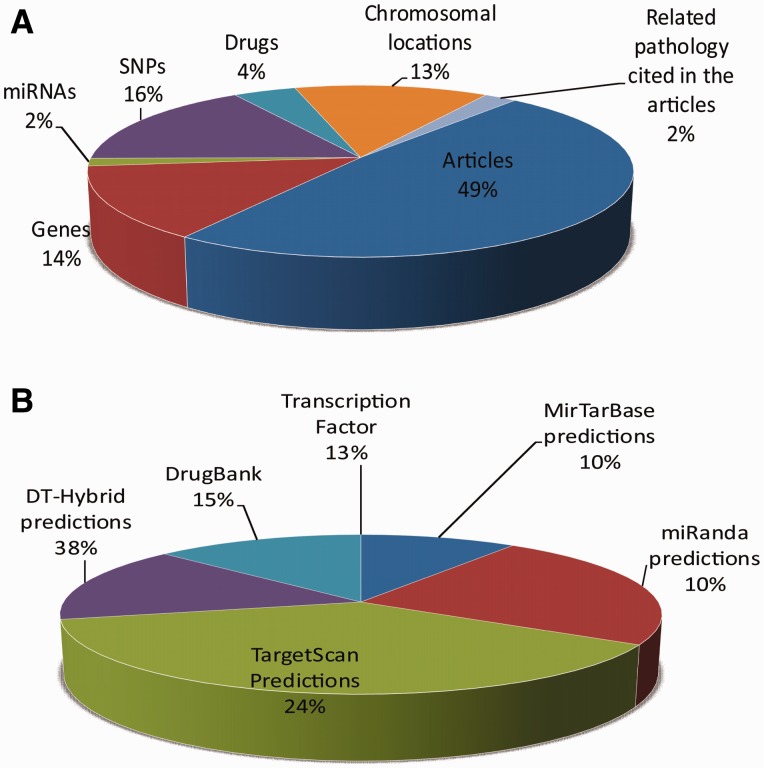
Statistics on (**A**) the manually curated data and (**B**) data obtained by using external sources and predictions software.

Statistics section reports the amount of data by category, with particular attention to the number of interactions by category. We refer to Figure 3 for the OCDB statistics. The number concerning the manually curated data are the following: 536 articles, 153 genes, 16 miRNAs, 180 SNPs, 48 drugs and 148 chromosomal locations. The data enriched by using external sources and predictions software are the following: 143 therapeutic indications from DrugBank, 50 pathways, 8701 gene and 566 miRNA trascription factor regulations, 37 435 MirTarBase predictions, 91 062 miRanda predictions, 146 125 TargetScan predictions, 59 218 DT-Hybrid predictions and 2276 DrugBank targeting relations.

#### Documentation.

The documentation section shows general information about the aim and the structure of OCDB, and a description of OCD.

The database is free for all users, and we distribute the data under a Creative Commons Attribution-NonCommercial-ShareAlike 3.0 License.

### Data maintenance

OCDB will be continuously updated, through manual screenings of new publications on PubMed (45) together with automatic procedures alerting new publications. Therefore, again the combination of manual and automatic procedures will extract and evaluate genetic information. After these steps OCDB will be updated.

In addition, researchers can suggest new or missing findings to be inserted in the database by contacting authors by email. Our contact information is reported in the ‘Contact’ page.

### Data distribution

#### Download/upload.

The whole database can be dumped, together with the SQL scripts, as a compressed archive.gz. Search results (i.e. by gene, miRNA, drug, article, SNP and region) can be downloaded in.txt or HTML format through the section ‘Info’. Data are also available for download in tabular format.

## Utility and discussion

Through the literature screening several inferences regarding genes and treatments about OCD may come to light. Until present day, most studies have been conducted on the same genes and the same drugs related to OCD. Some drugs used for the treatment of obsessions do not have a great efficacy in the treatment of OCD and are often themselves cause of the disease (i.e. Risperidone). According to the fact that in the worldwide population OCD is more common than it is really perceived (with a prevalence of 2–3%) (12), our system OCDB can help to give a comprehensive view about the main genes, drugs or miRNAs involved in the pathology. By querying OCDB, researchers can make new hypothesis and infer novel knowledge.

### Case study 1

*DRD2* (D(2) dopamine receptor) and *ADORA2A* (adenosine A2a receptor) a correlation among OCD caffeine, nicotine and SSRIs

#### Prior knowledge.

*DRD2* (D(2) dopamine receptor) is involved in OCD (64), and *ADORA2A* (adenosine A2a receptor) is a validated target site of hsa-miR339 associated with panic disorder (65). Caffeine can produce anxiogenic effect antagonizing adenosine at A(2A) receptors, which are colocalized with dopamine receptors in the brain (66). In a healthy person the effects are irrelevant or can be different if associated with polymorphisms in the *ADORA2A* gene (66).

#### How to use OCDB.

Through OCDB we may know which are the drugs or molecules active on *ADORA2A* and *DRD2.* This can be easily obtained by querying OCDB through the following path: Search *ADORA2A* (the same applies for *DRD2*), then click on ‘Drugs’ in the tab-bar menu, browse prediction data in the section ‘Drug-targets (DT-Hybrid)’ in OCDB. The results indicated that the molecules active on that genes are caffeine and nicotine while one of the drugs is fluvoxamine.

#### Posterior knowledge.

Pasquini *et al*. (67) present a case of OCD resistant to conventional treatments, which improved following nicotine augmentation administered as 4 mg chewing gum. Tizabi *et al*. (69) show as nicotine attenuates some symptoms of compulsive checking in a rat model of OCD.

#### Conclusion.

It could be interesting to investigate what effects the stimulation of these receptors produce in patients with anxiogenic background such as OCD or panic disorder as well as the simultaneous use of caffeine and nicotine with fluvoxamine.

### Case study 2

miRNAs prediction reporting on DRD2 and ADORA2A.

#### Prior knowledge.

It is known that *ADORA2A* (adenosine A2a receptor) is a validated target site of hsa-miR339 associated with panic disorder (65).

#### How to use OCDB.

OCDB stores also predicted miRNAs on *ADORA2A* (see Table 1). This may be visualized through the following path: Search *ADORA2A*, click ‘miRNAs’ in tab-bar menu of such gene and use prediction data in the section ‘miRNA target predictions (miRanda)’. The same must be done for *DRD2*. We have used the prediction data from miRanda (61), but there are also predictions from TargetScan (59) and MirTarBase (60).

**Table 1.** Association studies by using OCDB^a^

Genes	Predicted drugs (DT-Hybrid)	Predicted miRNAs (miRanda)	Description	Functional enrichment	References
**DRD2****^a^**	Caffeine, Nicotine, Fluvoxamine	miR-140-5p, miR-9, miR-150, miR-185, miR-190, miR-34c-5p, miR-377, miR-379, miR-330-5p, miR-326, miR-346, miR-449a, miR-181d, miR-539, miR-449b, miR-876-5p, miR-190b	No association reported in literature	Synaptic transmission, multicellular organismal signaling, transmission of nerve impulse, system process, neurological system process, neuron–neuron synaptic, **behavior**, regulation of synaptic transmission, regulation of transmission of nerve, regulation of neurological system, negative regulation of synaptic transmission, **learning or memory**, cell communication, startle response, response to drug, catecholamine transport, negative regulation of transmission of nerve impulse, drug binding, neurotransmitter receptor activity	Billett *et al.* (64)
**ADORA2A****^a^**	Caffeine, Nicotine, Fluvoxamine	miR-182; **miR-204**, **miR-211**, miR-214, miR-216a, miR-134, miR-149, miR-150, miR-34c-5p, miR-378, miR-383, miR-330-5p, miR-328, miR-342-3p, miR-326, **miR-339-5p**, miR-422a, **miR-485-5p**, **miR-488**, miR-494, miR-542-3p, miR-543, miR-34a	miR-488 is a repressor of POMC^c^ miR-339 associated with panic disorder^c^ miR-485-5p is associated with hoarding subtype of OCD^d^ miR-204 and miR-211 are associated with ALAD in social phobiae	Neurological system process, neuron–neuron synaptic, **behavior**, regulation of synaptic transmission, regulation of system process, regulation of transmission of nerve impulse, regulation of transmission of nerve impulse, signaling, startle response, response to drug, catecholamine transport, trans-membrane signaling receptor activity	Billett *et al*. (64) Muiños-Gimeno *et al*. (65) Muiños-Gimeno *et al*. (37) Donner *et al*. (69)

^a^Starting from a gene involved in OCD, DRD2 and ADORA2A, which is a validated target of hsa-miR339 associated with panic disorder, we found predicted drugs and miRNAs for these genes. A functional enrichment is also reported. ^b^Ref. (64). ^c^Ref. (65). ^d^Ref. (36). ^e^Ref. (69).

#### Posterior knowledge.

Among these, several miRNAs have been validated and predicted to be involved in OCD and panic disorder. For instance, miR-488 is a repressor of *POMC* (proopiomelanocortin) (65), miR-485-5p is associated with hoarding subtype of OCD (37), miR-204 and miR-211 are associated with *ALAD* (aminolevulinatedehydratase) in social phobia (69) and miR-339 is associated with panic disorder (65). In addition to having this data and using online resources, we have conducted a functional enrichment using WebGestalt (70) to individuate in which biological functions *DRD2* and *ADORA2A* are involved (see Table 1).

#### Conclusion.

The studies about the relation between miRNAs and OCD or some anxiety disorders are very recent. Only for few miRNAs, the function on this gene is known. Hence, the remaining predicted miRNAs could be interesting for further investigations (see Table 1).

### Case study 3

Polymorphisms in gene target, as in SLC6A, could influence treatments and the efficacy of drug therapies.

#### Prior knowledge.

Several OCD patients do not respond to conventional treatment. In OCD treatment, the main drug-targets are (i) SERT1 or 5-HTT solute carrier family 6 encoded by *SLC6A4*, (ii) 5-hydroxytryptamine (serotonin) receptor 2C- G protein-coupled encoded by *HTR2C* and (iii) dopamine transporter encoded by *SLC6A3*.

#### How to use OCDB.

OCDB contains SNPs within genes, miRNAs, miRNA-targets and drug-targets. The SNPs and the predicted drugs in *SLC6A4* can be obtained in OCDB through the following path: Search *SLC6A4,* click ‘SNP’ in tab-bar menu and browse the main SNPs associated with OCD in *SLC6A4*; also click ‘Drug’ in tab-bar menu and browse prediction data in the section ‘Drug-targets (DT-Hybrid). The predicted drugs are phentermine, pregabalin, doxycycline, venlafaxine, amitriptyline and tolcapone.

#### Conclusion.

An SNP within a drug-target could influence treatment and the efficacy of drug therapy. Studying predicted drug targets, researchers could make hypothesis on new drug combination for treatments.

### Case study 4

Finding a validation between oxidative stress and anxiety disorder.

#### Prior knowledge.

Another point of investigation involves the oxidative stress. *GSTP1* (glutathione S-transferase pi 1) is a target of clomipramine, a TCAs (71).

#### How to use OCDB.

We have interrogated OCDB to know which are other gene targets of Clomipramine. This can be easily obtained by querying OCDB through the following path: Search in drug section Clomipramine, by clicking ‘Genes’ in tab-bar menu. The list shows *GSTP1*, *HTR2A,* *HTR2B*, *HTR2C*, *SLC6A2* and *SLC6A4.* Then, we ask if each gene has glutathione as a predicted drug. Thus, by searching for each gene and clicking ‘Drugs’ in tab-bar menu, in the section ‘Drug target predictions (DT-Hydrid)’ we discovered that some of these genes are targets of semisynthetic molecules like glutathione: glutathione sulfonic acid, S-(P-nitrobenzyl)-glutathione and S-hexylglutathione, (*HTR2B*, *HTR2C*, *SLC6A2*, *SLC6A4*).

#### Posterior knowledge.

There is an interest on the oxidative stress in the etiology and progression and prevention of psychiatric disorders (72). Strong emotions or emotional stress can lead to mental states of depression or anxiety, with substantial consequences on lifestyle and health (73). During drug therapy, some miRNAs could change their concentration and thus influence the expression of their target. For example in a study on mice, miR-16 targets *SERT*. After treatment with fluoxetine, i.e. a SSRI (selective serotonin reuptake inhibitors), the miR-16 level increased with a reduced *SERT* expression. This is a case in which miRNAs play a synergistic role in therapy (74).

#### Conclusion.

The oxidative stress could alter and disrupt the neural circuits, which might be the implications of the use of glutathione in the treatment of OCD. In addition, miRNAs could have unexpected implications in this pathology, because miRNAs regulate multiple pathways in the brain in which minimal changes in gene expression or regulation could be fatal (76).

## Conclusion

OCDB is the first online resource containing genomic information related to OCD. We have collected the information from bibliography on PubMed. We have enriched the database with prediction analysis of miRNAs and drugs. Users can browse the information in the database by category (genes, articles, miRNAs, drugs, chromosomal region, SNP and drug target) or starting by searching a specific element (e.g. gene) and retrieve all connected information (targeting miRNAs, drugs and so on). By looking at OCDB, researchers can create a possible panel of genomic elements involved in the pathophysiology of disease. Finally, OCDB can provide the basis for further hypothesis and application of outcomes in the medium to long term.
